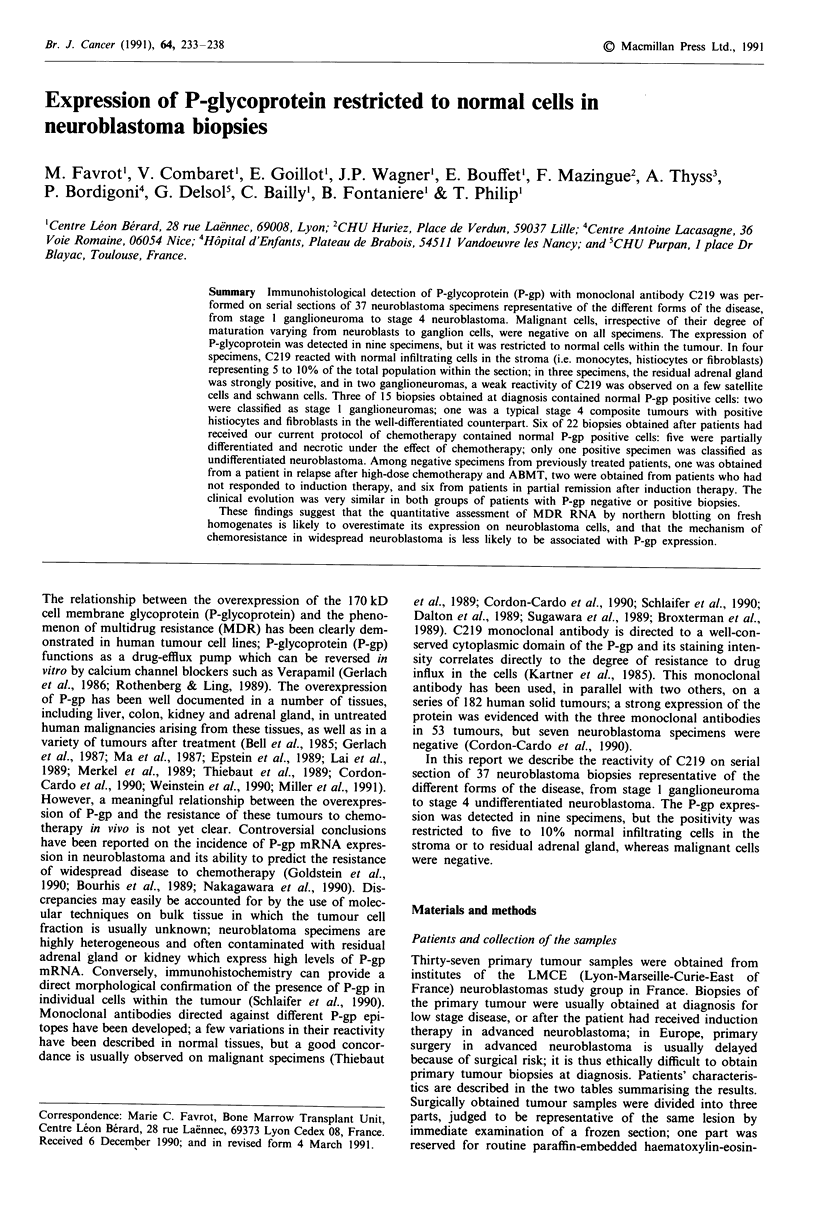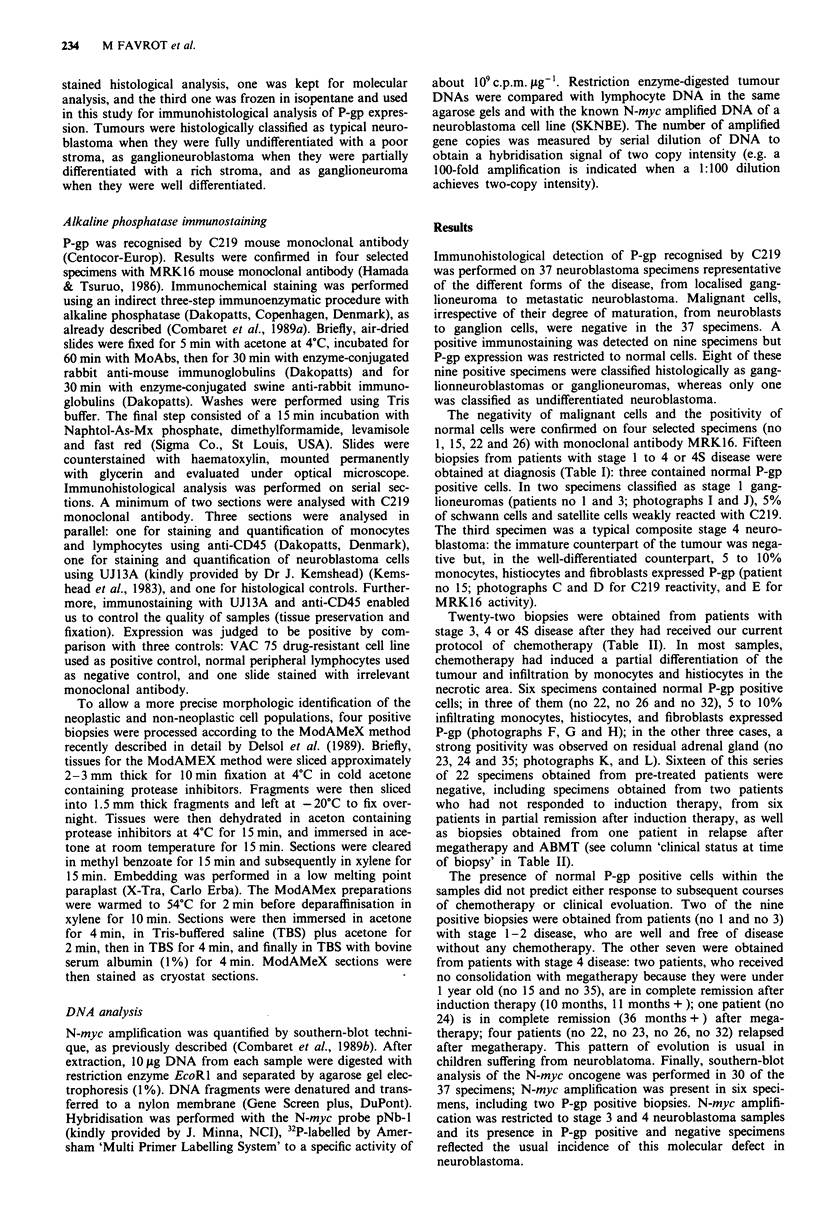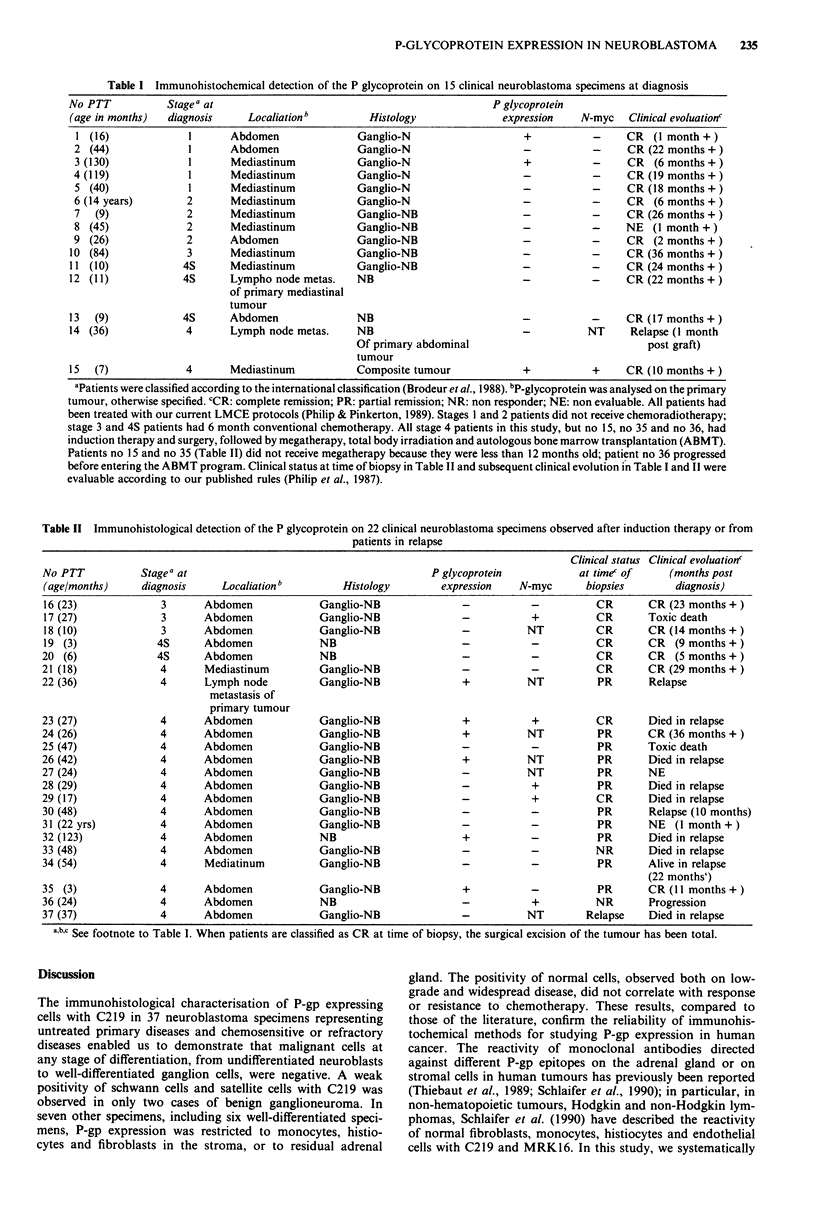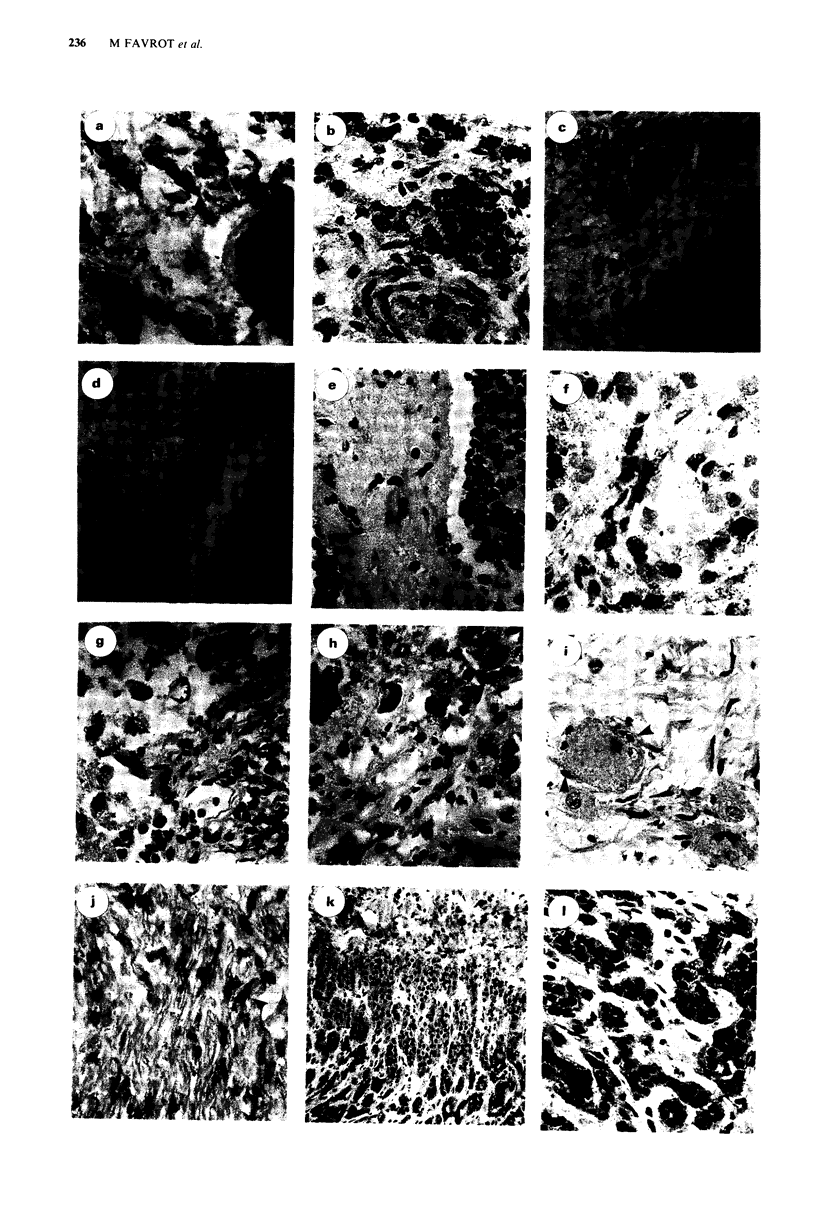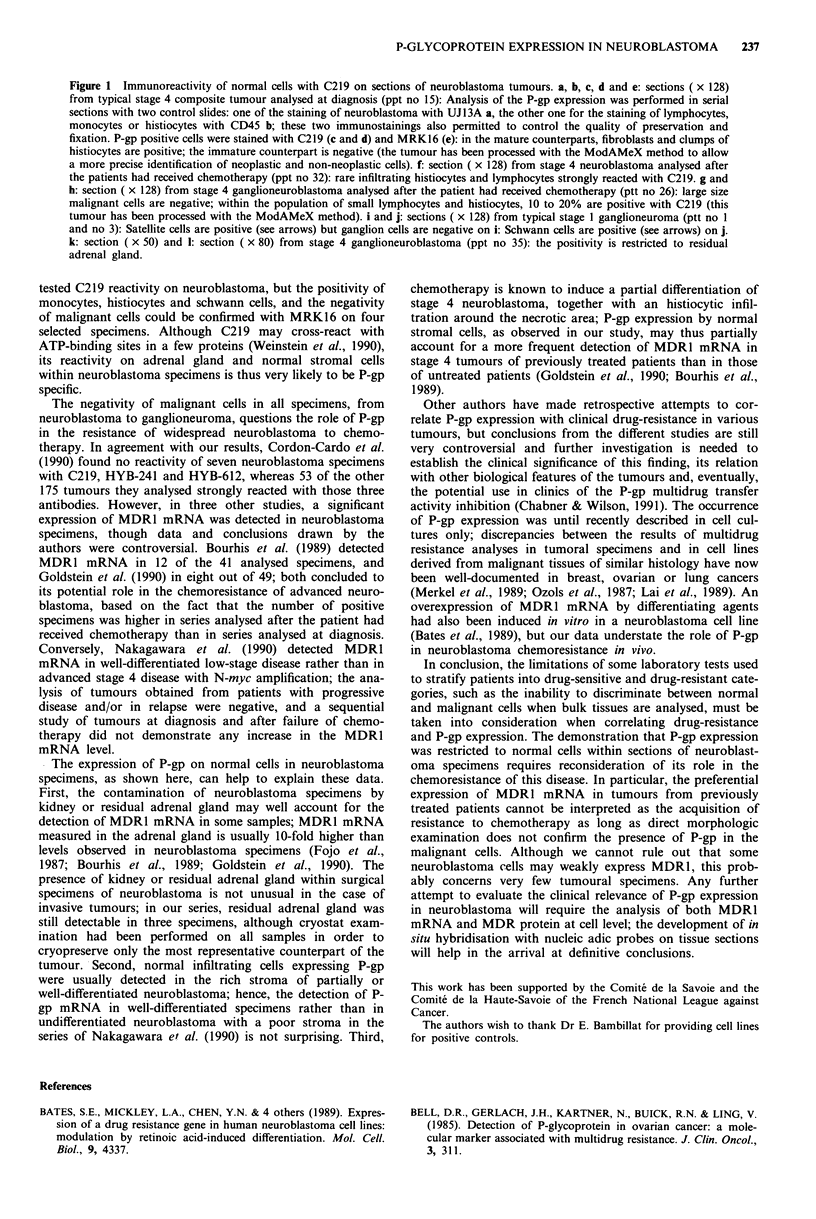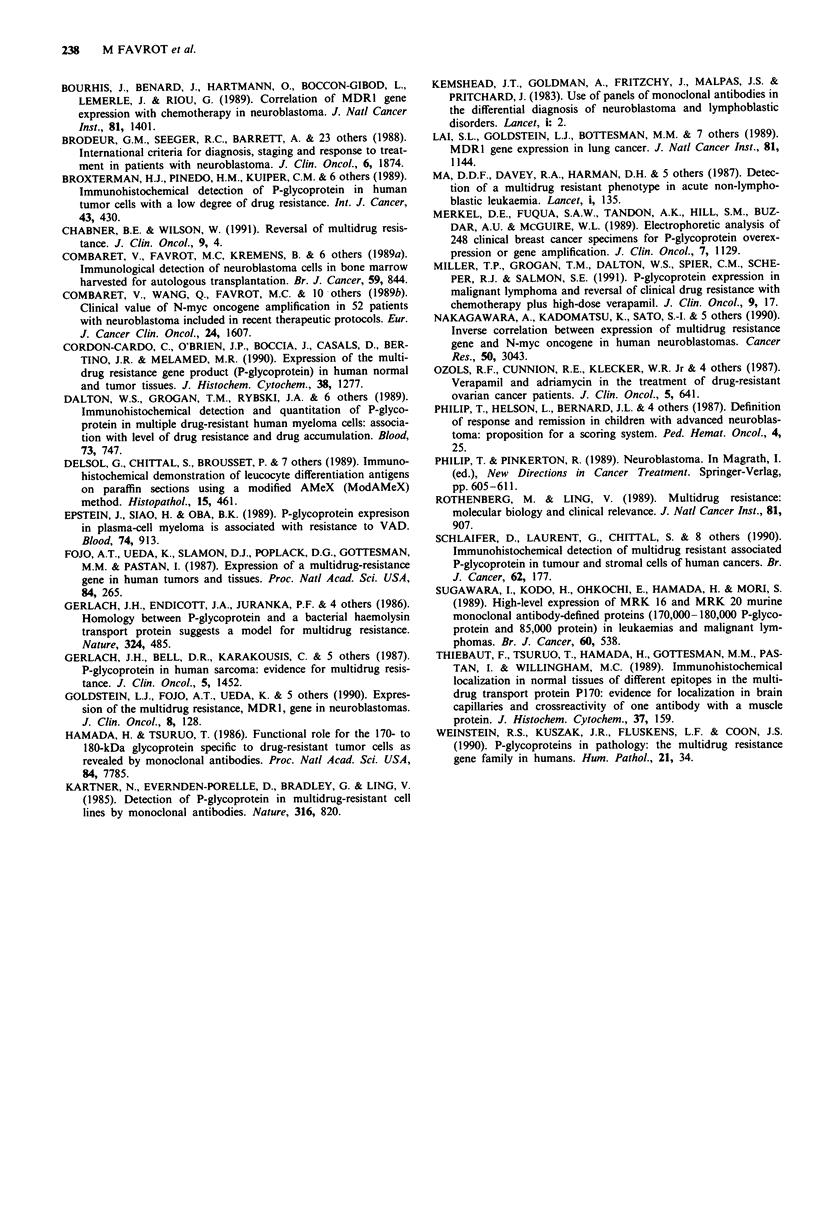# Expression of P-glycoprotein restricted to normal cells in neuroblastoma biopsies.

**DOI:** 10.1038/bjc.1991.282

**Published:** 1991-08

**Authors:** M. Favrot, V. Combaret, E. Goillot, J. P. Wagner, E. Bouffet, F. Mazingue, A. Thyss, P. Bordigoni, G. Delsol, C. Bailly

**Affiliations:** Centre Léon Bérard, Lyon, France.

## Abstract

**Images:**


Br. J. Cancer (1991), **64**, 233–238 © Macmillan Press Ltd., 1991

### Expression of P-glycoprotein restricted to normal cells in neuroblastoma biopsies

M. Favrot[1], V. Combaret[1], E. Goillot[1], J.P. Wagner[1], E. Bouffet[1], F. Mazingue[2], A. Thyss[3], P. Bordigoni[4], G. Delsol[5], C. Bailly[1], B. Fontaniere[1] & T. Philip[1]

[1]*Centre Léon Bérard, 28 rue Laënnec, 69008, Lyon;* [2]*CHU Huriez, Place de Verdun, 59037 Lille;* [4]*Centre Antoine Lacasagne, 36 Voie Romaine, 06054 Nice;* [4]*Hôpital d'Enfants, Plateau de Brabois, 54511 Vandoeuvre les Nancy; and* [5]*CHU Purpan, 1 place Dr Blayac, Toulouse, France.*

**Summary** Immunohistological detection of P-glycoprotein (P-gp) with monoclonal antibody C219 was performed on serial sections of 37 neuroblastoma specimens representative of the different forms of the disease, from stage 1 ganglioneuroma to stage 4 neuroblastoma. Malignant cells, irrespective of their degree of maturation varying from neuroblasts to ganglion cells, were negative on all specimens. The expression of P-glycoprotein was detected in nine specimens, but it was restricted to normal cells within the tumour. In four specimens, C219 reacted with normal infiltrating cells in the stroma (i.e. monocytes, histiocytes or fibroblasts) representing 5 to 10% of the total population within the section; in three specimens, the residual adrenal gland was strongly positive, and in two ganglioneuromas, a weak reactivity of C219 was observed on a few satellite cells and schwann cells. Three of 15 biopsies obtained at diagnosis contained normal P-gp positive cells: two were classified as stage 1 ganglioneuromas; one was a typical stage 4 composite tumours with positive histiocytes and fibroblasts in the well-differentiated counterpart. Six of 22 biopsies obtained after patients had received our current protocol of chemotherapy contained normal P-gp positive cells: five were partially differentiated and necrotic under the effect of chemotherapy; only one positive specimen was classified as undifferentiated neuroblastoma. Among negative specimens from previously treated patients, one was obtained from a patient in relapse after high-dose chemotherapy and ABMT, two were obtained from patients who had not responded to induction therapy, and six from patients in partial remission after induction therapy. The clinical evolution was very similar in both groups of patients with P-gp negative or positive biopsies.

These findings suggest that the quantitative assessment of MDR RNA by northern blotting on fresh homogenates is likely to overestimate its expression on neuroblastoma cells, and that the mechanism of chemoresistance in widespread neuroblastoma is less likely to be associated with P-gp expression.

The relationship between the overexpression of the 170 kD cell membrane glycoprotein (P-glycoprotein) and the phenomenon of multidrug resistance (MDR) has been clearly demonstrated in human tumour cell lines; P-glycoprotein (P-gp) functions as a drug-efflux pump which can be reversed *in vitro* by calcium channel blockers such as Verapamil (Gerlach *et al.*, 1986; Rothenberg & Ling, 1989). The overexpression of P-gp has been well documented in a number of tissues, including liver, colon, kidney and adrenal gland, in untreated human malignancies arising from these tissues, as well as in a variety of tumours after treatment (Bell *et al.*, 1985; Gerlach *et al.*, 1987; Ma *et al.*, 1987; Epstein *et al.*, 1989; Lai *et al.*, 1989; Merkel *et al.*, 1989; Thiebaut *et al.*, 1989; Cordon-Cardo *et al.*, 1990; Weinstein *et al.*, 1990; Miller *et al.*, 1991). However, a meaningful relationship between the overexpression of P-gp and the resistance of these tumours to chemotherapy *in vivo* is not yet clear. Controversial conclusions have been reported on the incidence of P-gp mRNA expression in neuroblastoma and its ability to predict the resistance of widespread disease to chemotherapy (Goldstein *et al.*, 1990; Bourhis *et al.*, 1989; Nakagawara *et al.*, 1990). Discrepancies may easily be accounted for by the use of molecular techniques on bulk tissue in which the tumour cell fraction is usually unknown; neuroblatoma specimens are highly heterogeneous and often contaminated with residual adrenal gland or kidney which express high levels of P-gp mRNA. Conversely, immunohistochemistry can provide a direct morphological confirmation of the presence of P-gp in individual cells within the tumour (Schlaifer *et al.*, 1990). Monoclonal antibodies directed against different P-gp epitopes have been developed; a few variations in their reactivity have been described in normal tissues, but a good concordance is usually observed on malignant specimens (Thiebaut *et al.*, 1989; Cordon-Cardo *et al.*, 1990; Schlaifer *et al.*, 1990; Dalton *et al.*, 1989; Sugawara *et al.*, 1989; Broxterman *et al.*, 1989). C219 monoclonal antibody is directed to a well-conserved cytoplasmic domain of the P-gp and its staining intensity correlates directly to the degree of resistance to drug influx in the cells (Kartner *et al.*, 1985). This monoclonal antibody has been used, in parallel with two others, on a series of 182 human solid tumours; a strong expression of the protein was evidenced with the three monoclonal antibodies in 53 tumours, but seven neuroblastoma specimens were negative (Cordon-Cardo *et al.*, 1990).

In this report we describe the reactivity of C219 on serial section of 37 neuroblastoma biopsies representative of the different forms of the disease, from stage 1 ganglioneuroma to stage 4 undifferentiated neuroblastoma. The P-gp expression was detected in nine specimens, but the positivity was restricted to five to 10% normal infiltrating cells in the stroma or to residual adrenal gland, whereas malignant cells were negative.

### Materials and methods

### *Patients and collection of the samples*

Thirty-seven primary tumour samples were obtained from institutes of the LMCE (Lyon-Marseille-Curie-East of France) neuroblastomas study group in France. Biopsies of the primary tumour were usually obtained at diagnosis for low stage disease, or after the patient had received induction therapy in advanced neuroblastoma; in Europe, primary surgery in advanced neuroblastoma is usually delayed because of surgical risk; it is thus ethically difficult to obtain primary tumour biopsies at diagnosis. Patients' characteristics are described in the two tables summarising the results. Surgically obtained tumour samples were divided into three parts, judged to be representative of the same lesion by immediate examination of a frozen section; one part was reserved for routine paraffin-embedded haematoxylin-eosin-

Correspondence: Marie C. Favrot, Bone Marrow Transplant Unit, Centre Léon Bérard, 28 rue Laënnec, 69373 Lyon Cedex 08, France.
Received 6 December 1990; and in revised form 4 March 1991.

stained histological analysis, one was kept for molecular analysis, and the third one was frozen in isopentane and used in this study for immunohistological analysis of P-gp expression. Tumours were histologically classified as typical neuroblastoma when they were fully undifferentiated with a poor stroma, as ganglioneuroblastoma when they were partially differentiated with a rich stroma, and as ganglioneuroma when they were well differentiated.

*Alkaline phosphatase immunostaining*

P-gp was recognised by C219 mouse monoclonal antibody (Centocor-Europ). Results were confirmed in four selected specimens with MRK16 mouse monoclonal antibody (Hamada & Tsuruo, 1986). Immunochemical staining was performed using an indirect three-step immunoenzymatic procedure with alkaline phosphatase (Dakopatts, Copenhagen, Denmark), as already described (Combaret *et al.*, 1989*a*). Briefly, air-dried slides were fixed for 5 min with acetone at 4°C, incubated for 60 min with MoAbs, then for 30 min with enzyme-conjugated rabbit anti-mouse immunoglobulins (Dakopatts) and for 30 min with enzyme-conjugated swine anti-rabbit immunoglobulins (Dakopatts). Washes were performed using Tris buffer. The final step consisted of a 15 min incubation with Naphtol-As-Mx phosphate, dimethylformamide, levamisole and fast red (Sigma Co., St Louis, USA). Slides were counterstained with haematoxylin, mounted permanently with glycerin and evaluated under optical microscope. Immunohistological analysis was performed on serial sections. A minimum of two sections were analysed with C219 monoclonal antibody. Three sections were analysed in parallel: one for staining and quantification of monocytes and lymphocytes using anti-CD45 (Dakopatts, Denmark), one for staining and quantification of neuroblastoma cells using UJ13A (kindly provided by Dr J. Kemshead) (Kemshead *et al.*, 1983), and one for histological controls. Furthermore, immunostaining with UJ13A and anti-CD45 enabled us to control the quality of samples (tissue preservation and fixation). Expression was judged to be positive by comparison with three controls: VAC 75 drug-resistant cell line used as positive control, normal peripheral lymphocytes used as negative control, and one slide stained with irrelevant monoclonal antibody.

To allow a more precise morphologic identification of the neoplastic and non-neoplastic cell populations, four positive biopsies were processed according to the ModAMeX method recently described in detail by Delsol *et al.* (1989). Briefly, tissues for the ModAMEX method were sliced approximately 2–3 mm thick for 10 min fixation at 4°C in cold acetone containing protease inhibitors. Fragments were then sliced into 1.5 mm thick fragments and left at −20°C to fix overnight. Tissues were then dehydrated in aceton containing protease inhibitors at 4°C for 15 min, and immersed in acetone at room temperature for 15 min. Sections were cleared in methyl benzoate for 15 min and subsequently in xylene for 15 min. Embedding was performed in a low melting point paraplast (X-Tra, Carlo Erba). The ModAMex preparations were warmed to 54°C for 2 min before deparaffinisation in xylene for 10 min. Sections were then immersed in acetone for 4 min, in Tris-buffered saline (TBS) plus acetone for 2 min, then in TBS for 4 min, and finally in TBS with bovine serum albumin (1%) for 4 min. ModAMeX sections were then stained as cryostat sections.

*DNA analysis*

N-*myc* amplification was quantified by southern-blot technique, as previously described (Combaret *et al.*, 1989*b*). After extraction, 10 μg DNA from each sample were digested with restriction enzyme *Eco*R1 and separated by agarose gel electrophoresis (1%). DNA fragments were denatured and transferred to a nylon membrane (Gene Screen plus, DuPont). Hybridisation was performed with the N-*myc* probe pNb-1 (kindly provided by J. Minna, NCI), $^{32}$P-labelled by Amersham 'Multi Primer Labelling System' to a specific activity of about $10^9$ c.p.m. $\mu g^{-1}$. Restriction enzyme-digested tumour DNAs were compared with lymphocyte DNA in the same agarose gels and with the known N-*myc* amplified DNA of a neuroblastoma cell line (SKNBE). The number of amplified gene copies was measured by serial dilution of DNA to obtain a hybridisation signal of two copy intensity (e.g. a 100-fold amplification is indicated when a 1:100 dilution achieves two-copy intensity).

### Results

Immunohistological detection of P-gp recognised by C219 was performed on 37 neuroblastoma specimens representative of the different forms of the disease, from localised ganglioneuroma to metastatic neuroblastoma. Malignant cells, irrespective of their degree of maturation, from neuroblasts to ganglion cells, were negative in the 37 specimens. A positive immunostaining was detected on nine specimens but P-gp expression was restricted to normal cells. Eight of these nine positive specimens were classified histologically as ganglionneuroblastomas or ganglioneuromas, whereas only one was classified as undifferentiated neuroblastoma.

The negativity of malignant cells and the positivity of normal cells were confirmed on four selected specimens (no 1, 15, 22 and 26) with monoclonal antibody MRK16. Fifteen biopsies from patients with stage 1 to 4 or 4S disease were obtained at diagnosis (Table I): three contained normal P-gp positive cells. In two specimens classified as stage 1 ganglioneuromas (patients no 1 and 3; photographs I and J), 5% of schwann cells and satellite cells weakly reacted with C219. The third specimen was a typical composite stage 4 neuroblastoma: the immature counterpart of the tumour was negative but, in the well-differentiated counterpart, 5 to 10% monocytes, histiocytes and fibroblasts expressed P-gp (patient no 15; photographs C and D for C219 reactivity, and E for MRK16 activity).

Twenty-two biopsies were obtained from patients with stage 3, 4 or 4S disease after they had received our current protocol of chemotherapy (Table II). In most samples, chemotherapy had induced a partial differentiation of the tumour and infiltration by monocytes and histiocytes in the necrotic area. Six specimens contained normal P-gp positive cells; in three of them (no 22, no 26 and no 32), 5 to 10% infiltrating monocytes, histiocytes, and fibroblasts expressed P-gp (photographs F, G and H); in the other three cases, a strong positivity was observed on residual adrenal gland (no 23, 24 and 35; photographs K, and L). Sixteen of this series of 22 specimens obtained from pre-treated patients were negative, including specimens obtained from two patients who had not responded to induction therapy, from six patients in partial remission after induction therapy, as well as biopsies obtained from one patient in relapse after megatherapy and ABMT (see column 'clinical status at time of biopsy' in Table II).

The presence of normal P-gp positive cells within the samples did not predict either response to subsequent courses of chemotherapy or clinical evoluation. Two of the nine positive biopsies were obtained from patients (no 1 and no 3) with stage 1–2 disease, who are well and free of disease without any chemotherapy. The other seven were obtained from patients with stage 4 disease: two patients, who received no consolidation with megatherapy because they were under 1 year old (no 15 and no 35), are in complete remission after induction therapy (10 months, 11 months + ); one patient (no 24) is in complete remission (36 months + ) after megatherapy; four patients (no 22, no 23, no 26, no 32) relapsed after megatherapy. This pattern of evolution is usual in children suffering from neuroblatoma. Finally, southern-blot analysis of the N-*myc* oncogene was performed in 30 of the 37 specimens; N-*myc* amplification was present in six specimens, including two P-gp positive biopsies. N-*myc* amplification was restricted to stage 3 and 4 neuroblastoma samples and its presence in P-gp positive and negative specimens reflected the usual incidence of this molecular defect in neuroblastoma.

**Table I** Immunohistochemical detection of the P glycoprotein on 15 clinical neuroblastoma specimens at diagnosis

| *No PTT (age in months)* | *Stage[a] at diagnosis* | *Localiation[b]* | *Histology* | *P glycoprotein expression* | *N*-myc | *Clinical evoluation[c]* |
|---|---|---|---|---|---|---|
| 1 (16) | 1 | Abdomen | Ganglio-N | + | – | CR (1 month + ) |
| 2 (44) | 1 | Abdomen | Ganglio-N | – | – | CR (22 months + ) |
| 3 (130) | 1 | Mediastinum | Ganglio-N | + | – | CR (6 months + ) |
| 4 (119) | 1 | Mediastinum | Ganglio-N | – | – | CR (19 months + ) |
| 5 (40) | 1 | Mediastinum | Ganglio-N | – | – | CR (18 months + ) |
| 6 (14 years) | 2 | Mediastinum | Ganglio-N | – | – | CR (6 months + ) |
| 7 (9) | 2 | Mediastinum | Ganglio-NB | – | – | CR (26 months + ) |
| 8 (45) | 2 | Mediastinum | Ganglio-NB | – | – | NE (1 month + ) |
| 9 (26) | 2 | Abdomen | Ganglio-NB | – | – | CR (2 months + ) |
| 10 (84) | 3 | Mediastinum | Ganglio-NB | – | – | CR (36 months + ) |
| 11 (10) | 4S | Mediastinum | Ganglio-NB | – | – | CR (24 months + ) |
| 12 (11) | 4S | Lympho node metas. of primary mediastinal tumour | NB | – | – | CR (22 months + ) |
| 13 (9) | 4S | Abdomen | NB | – | – | CR (17 months + ) |
| 14 (36) | 4 | Lymph node metas. | NB Of primary abdominal tumour | – | NT | Relapse (1 month post graft) |
| 15 (7) | 4 | Mediastinum | Composite tumour | + | + | CR (10 months + ) |

[a]Patients were classified according to the international classification (Brodeur *et al.*, 1988). [b]P-glycoprotein was analysed on the primary tumour, otherwise specified. [c]CR: complete remission; PR: partial remission; NR: non responder; NE: non evaluable. All patients had been treated with our current LMCE protocols (Philip & Pinkerton, 1989). Stages 1 and 2 patients did not receive chemoradiotherapy; stage 3 and 4S patients had 6 month conventional chemotherapy. All stage 4 patients in this study, but no 15, no 35 and no 36, had induction therapy and surgery, followed by megatherapy, total body irradiation and autologous bone marrow transplantation (ABMT). Patients no 15 and no 35 (Table II) did not receive megatherapy because they were less than 12 months old; patient no 36 progressed before entering the ABMT program. Clinical status at time of biopsy in Table II and subsequent clinical evolution in Table I and II were evaluable according to our published rules (Philip *et al.*, 1987).

**Table II** Immunohistological detection of the P glycoprotein on 22 clinical neuroblastoma specimens observed after induction therapy or from patients in relapse

| *No PTT (age/months)* | *Stage[a] at diagnosis* | *Localiation[b]* | *Histology* | *P glycoprotein expression* | *N*-myc | *Clinical status at time[c] of biopsies* | *Clinical evoluation[c] (months post diagnosis)* |
|---|---|---|---|---|---|---|---|
| 16 (23) | 3 | Abdomen | Ganglio-NB | – | – | CR | CR (23 months + ) |
| 17 (27) | 3 | Abdomen | Ganglio-NB | – | + | CR | Toxic death |
| 18 (10) | 3 | Abdomen | Ganglio-NB | – | NT | CR | CR (14 months + ) |
| 19 (3) | 4S | Abdomen | NB | – | – | CR | CR (9 months + ) |
| 20 (6) | 4S | Abdomen | NB | – | – | CR | CR (5 months + ) |
| 21 (18) | 4 | Mediastinum | Ganglio-NB | – | – | CR | CR (29 months + ) |
| 22 (36) | 4 | Lymph node metastasis of primary tumour | Ganglio-NB | + | NT | PR | Relapse |
| 23 (27) | 4 | Abdomen | Ganglio-NB | + | + | CR | Died in relapse |
| 24 (26) | 4 | Abdomen | Ganglio-NB | + | NT | PR | CR (36 months + ) |
| 25 (47) | 4 | Abdomen | Ganglio-NB | – | – | PR | Toxic death |
| 26 (42) | 4 | Abdomen | Ganglio-NB | + | NT | PR | Died in relapse |
| 27 (24) | 4 | Abdomen | Ganglio-NB | – | NT | PR | NE |
| 28 (29) | 4 | Abdomen | Ganglio-NB | – | + | PR | Died in relapse |
| 29 (17) | 4 | Abdomen | Ganglio-NB | – | + | CR | Died in relapse |
| 30 (48) | 4 | Abdomen | Ganglio-NB | – | – | PR | Relapse (10 months) |
| 31 (22 yrs) | 4 | Abdomen | Ganglio-NB | – | – | PR | NE (1 month + ) |
| 32 (123) | 4 | Abdomen | NB | + | – | PR | Died in relapse |
| 33 (48) | 4 | Abdomen | Ganglio-NB | – | – | NR | Died in relapse |
| 34 (54) | 4 | Mediatinum | Ganglio-NB | – | – | PR | Alive in relapse (22 months[c]) |
| 35 (3) | 4 | Abdomen | Ganglio-NB | + | – | PR | CR (11 months + ) |
| 36 (24) | 4 | Abdomen | NB | – | + | NR | Progression |
| 37 (37) | 4 | Abdomen | Ganglio-NB | – | NT | Relapse | Died in relapse |

[a,b,c] See footnote to Table I. When patients are classified as CR at time of biopsy, the surgical excision of the tumour has been total.

### Discussion

The immunohistological characterisation of P-gp expressing cells with C219 in 37 neuroblastoma specimens representing untreated primary diseases and chemosensitive or refractory diseases enabled us to demonstrate that malignant cells at any stage of differentiation, from undifferentiated neuroblasts to well-differentiated ganglion cells, were negative. A weak positivity of schwann cells and satellite cells with C219 was observed in only two cases of benign ganglioneuroma. In seven other specimens, including six well-differentiated specimens, P-gp expression was restricted to monocytes, histiocytes and fibroblasts in the stroma, or to residual adrenal gland. The positivity of normal cells, observed both on low-grade and widespread disease, did not correlate with response or resistance to chemotherapy. These results, compared to those of the literature, confirm the reliability of immunohistochemical methods for studying P-gp expression in human cancer. The reactivity of monoclonal antibodies directed against different P-gp epitopes on the adrenal gland or on stromal cells in human tumours has previously been reported (Thiebaut *et al.*, 1989; Schlaifer *et al.*, 1990); in particular, in non-hematopoietic tumours, Hodgkin and non-Hodgkin lymphomas, Schlaifer *et al.* (1990) have described the reactivity of normal fibroblasts, monocytes, histiocytes and endothelial cells with C219 and MRK16. In this study, we systematically a
b
c
d
e
f
g
h
i
j
k
l

**Figure 1** Immunoreactivity of normal cells with C219 on sections of neuroblastoma tumours. **a**, **b**, **c**, **d** and **e**: sections ( × 128) from typical stage 4 composite tumour analysed at diagnosis (ppt no 15): Analysis of the P-gp expression was performed in serial sections with two control slides: one of the staining of neuroblastoma with UJ13A **a**, the other one for the staining of lymphocytes, monocytes or histiocytes with CD45 **b**; these two immunostainings also permitted to control the quality of preservation and fixation. P-gp positive cells were stained with C219 (**c** and **d**) and MRK16 (**e**): in the mature counterparts, fibroblasts and clumps of histiocytes are positive; the immature counterpart is negative (the tumour has been processed with the ModAMeX method to allow a more precise identification of neoplastic and non-neoplastic cells). **f**: section ( × 128) from stage 4 neuroblastoma analysed after the patients had received chemotherapy (ppt no 32): rare infiltrating histiocytes and lymphocytes strongly reacted with C219. **g** and **h**: section ( × 128) from stage 4 ganglioneuroblastoma analysed after the patient had received chemotherapy (ptt no 26): large size malignant cells are negative; within the population of small lymphocytes and histiocytes, 10 to 20% are positive with C219 (this tumour has been processed with the ModAMeX method). **i** and **j**: sections ( × 128) from typical stage 1 ganglioneuroma (ptt no 1 and no 3): Satellite cells are positive (see arrows) but ganglion cells are negative on **i**: Schwann cells are positive (see arrows) on **j**. **k**: section ( × 50) and **l**: section ( × 80) from stage 4 ganglioneuroblastoma (ppt no 35): the positivity is restricted to residual adrenal gland.

tested C219 reactivity on neuroblastoma, but the positivity of monocytes, histiocytes and schwann cells, and the negativity of malignant cells could be confirmed with MRK16 on four selected specimens. Although C219 may cross-react with ATP-binding sites in a few proteins (Weinstein *et al.*, 1990), its reactivity on adrenal gland and normal stromal cells within neuroblastoma specimens is thus very likely to be P-gp specific.

The negativity of malignant cells in all specimens, from neuroblastoma to ganglioneuroma, questions the role of P-gp in the resistance of widespread neuroblastoma to chemotherapy. In agreement with our results, Cordon-Cardo *et al.* (1990) found no reactivity of seven neuroblastoma specimens with C219, HYB-241 and HYB-612, whereas 53 of the other 175 tumours they analysed strongly reacted with those three antibodies. However, in three other studies, a significant expression of MDR1 mRNA was detected in neuroblastoma specimens, though data and conclusions drawn by the authors were controversial. Bourhis *et al.* (1989) detected MDR1 mRNA in 12 of the 41 analysed specimens, and Goldstein *et al.* (1990) in eight out of 49; both concluded to its potential role in the chemoresistance of advanced neuroblastoma, based on the fact that the number of positive specimens was higher in series analysed after the patient had received chemotherapy than in series analysed at diagnosis. Conversely, Nakagawara *et al.* (1990) detected MDR1 mRNA in well-differentiated low-stage disease rather than in advanced stage 4 disease with N-*myc* amplification; the analysis of tumours obtained from patients with progressive disease and/or in relapse were negative, and a sequential study of tumours at diagnosis and after failure of chemotherapy did not demonstrate any increase in the MDR1 mRNA level.

The expression of P-gp on normal cells in neuroblastoma specimens, as shown here, can help to explain these data. First, the contamination of neuroblastoma specimens by kidney or residual adrenal gland may well account for the detection of MDR1 mRNA in some samples; MDR1 mRNA measured in the adrenal gland is usually 10-fold higher than levels observed in neuroblastoma specimens (Fojo *et al.*, 1987; Bourhis *et al.*, 1989; Goldstein *et al.*, 1990). The presence of kidney or residual adrenal gland within surgical specimens of neuroblastoma is not unusual in the case of invasive tumours; in our series, residual adrenal gland was still detectable in three specimens, although cryostat examination had been performed on all samples in order to cryopreserve only the most representative counterpart of the tumour. Second, normal infiltrating cells expressing P-gp were usually detected in the rich stroma of partially or well-differentiated neuroblastoma; hence, the detection of P-gp mRNA in well-differentiated specimens rather than in undifferentiated neuroblastoma with a poor stroma in the series of Nakagawara *et al.* (1990) is not surprising. Third, chemotherapy is known to induce a partial differentiation of stage 4 neuroblastoma, together with an histiocytic infiltration around the necrotic area; P-gp expression by normal stromal cells, as observed in our study, may thus partially account for a more frequent detection of MDR1 mRNA in stage 4 tumours of previously treated patients than in those of untreated patients (Goldstein *et al.*, 1990; Bourhis *et al.*, 1989).

Other authors have made retrospective attempts to correlate P-gp expression with clinical drug-resistance in various tumours, but conclusions from the different studies are still very controversial and further investigation is needed to establish the clinical significance of this finding, its relation with other biological features of the tumours and, eventually, the potential use in clinics of the P-gp multidrug transfer activity inhibition (Chabner & Wilson, 1991). The occurrence of P-gp expression was until recently described in cell cultures only; discrepancies between the results of multidrug resistance analyses in tumoral specimens and in cell lines derived from malignant tissues of similar histology have now been well-documented in breast, ovarian or lung cancers (Merkel *et al.*, 1989; Ozols *et al.*, 1987; Lai *et al.*, 1989). An overexpression of MDR1 mRNA by differentiating agents had also been induced *in vitro* in a neuroblastoma cell line (Bates *et al.*, 1989), but our data understate the role of P-gp in neuroblastoma chemoresistance *in vivo*.

In conclusion, the limitations of some laboratory tests used to stratify patients into drug-sensitive and drug-resistant categories, such as the inability to discriminate between normal and malignant cells when bulk tissues are analysed, must be taken into consideration when correlating drug-resistance and P-gp expression. The demonstration that P-gp expression was restricted to normal cells within sections of neuroblastoma specimens requires reconsideration of its role in the chemoresistance of this disease. In particular, the preferential expression of MDR1 mRNA in tumours from previously treated patients cannot be interpreted as the acquisition of resistance to chemotherapy as long as direct morphologic examination does not confirm the presence of P-gp in the malignant cells. Although we cannot rule out that some neuroblastoma cells may weakly express MDR1, this probably concerns very few tumoural specimens. Any further attempt to evaluate the clinical relevance of P-gp expression in neuroblastoma will require the analysis of both MDR1 mRNA and MDR protein at cell level; the development of *in situ* hybridisation with nucleic adic probes on tissue sections will help in the arrival at definitive conclusions.

This work has been supported by the Comité de la Savoie and the Comité de la Haute-Savoie of the French National League against Cancer.

The authors wish to thank Dr E. Bambillat for providing cell lines for positive controls.

### References

BATES, S.E., MICKLEY, L.A., CHEN, Y.N. & 4 others (1989). Expression of a drug resistance gene in human neuroblastoma cell lines: modulation by retinoic acid-induced differentiation. *Mol. Cell. Biol.*, **9**, 4337.

BELL, D.R., GERLACH, J.H., KARTNER, N., BUICK, R.N. & LING, V. (1985). Detection of P-glycoprotein in ovarian cancer: a molecular marker associated with multidrug resistance. *J. Clin. Oncol.*, **3**, 311.

BOURHIS, J., BENARD, J., HARTMANN, O., BOCCON-GIBOD, L., LEMERLE, J. & RIOU, G. (1989). Correlation of MDR1 gene expression with chemotherapy in neuroblastoma. *J. Natl Cancer Inst.*, **81**, 1401.

BRODEUR, G.M., SEEGER, R.C., BARRETT, A. & 23 others (1988). International criteria for diagnosis, staging and response to treatment in patients with neuroblastoma. *J. Clin. Oncol.*, **6**, 1874.

BROXTERMAN, H.J., PINEDO, H.M., KUIPER, C.M. & 6 others (1989). Immunohistochemical detection of P-glycoprotein in human tumor cells with a low degree of drug resistance. *Int. J. Cancer*, **43**, 430.

CHABNER, B.E. & WILSON, W. (1991). Reversal of multidrug resistance. *J. Clin. Oncol.*, **9**, 4.

COMBARET, V., FAVROT, M.C, KREMENS, B. & 6 others (1989*a*). Immunological detection of neuroblastoma cells in bone marrow harvested for autologous transplantation. *Br. J. Cancer*, **59**, 844.

COMBARET, V., WANG, Q., FAVROT, M.C. & 10 others (1989*b*). Clinical value of N-myc oncogene amplification in 52 patients with neuroblastoma included in recent therapeutic protocols. *Eur. J. Cancer Clin. Oncol.*, **24**, 1607.

CORDON-CARDO, C., O'BRIEN, J.P., BOCCIA, J., CASALS, D., BERTINO, J.R. & MELAMED, M.R. (1990). Expression of the multidrug resistance gene product (P-glycoprotein) in human normal and tumor tissues. *J. Histochem. Cytochem.*, **38**, 1277.

DALTON, W.S., GROGAN, T.M., RYBSKI, J.A. & 6 others (1989). Immunohistochemical detection and quantitation of P-glycoprotein in multiple drug-resistant human myeloma cells: association with level of drug resistance and drug accumulation. *Blood*, **73**, 747.

DELSOL, G., CHITTAL, S., BROUSSET, P. & 7 others (1989). Immunohistochemical demonstration of leucocyte differentiation antigens on paraffin sections using a modified AMeX (ModAMeX) method. *Histopathol.*, **15**, 461.

EPSTEIN, J., SIAO, H. & OBA, B.K. (1989). P-glycoprotein expresison in plasma-cell myeloma is associated with resistance to VAD. *Blood*, **74**, 913.

FOJO, A.T., UEDA, K., SLAMON, D.J., POPLACK, D.G., GOTTESMAN, M.M. & PASTAN, I. (1987). Expression of a multidrug-resistance gene in human tumors and tissues. *Proc. Natl Acad. Sci. USA*, **84**, 265.

GERLACH, J.H., ENDICOTT, J.A., JURANKA, P.F. & 4 others (1986). Homology between P-glycoprotein and a bacterial haemolysin transport protein suggests a model for multidrug resistance. *Nature*, **324**, 485.

GERLACH, J.H., BELL, D.R., KARAKOUSIS, C. & 5 others (1987). P-glycoprotein in human sarcoma: evidence for multidrug resistance. *J. Clin. Oncol.*, **5**, 1452.

GOLDSTEIN, L.J., FOJO, A.T., UEDA, K. & 5 others (1990). Expression of the multidrug resistance, MDR1, gene in neuroblastomas. *J. Clin. Oncol.*, **8**, 128.

HAMADA, H. & TSURUO, T. (1986). Functional role for the 170- to 180-kDa glycoprotein specific to drug-resistant tumor cells as revealed by monoclonal antibodies. *Proc. Natl Acad. Sci. USA*, **84**, 7785.

KARTNER, N., EVERNDEN-PORELLE, D., BRADLEY, G. & LING, V. (1985). Detection of P-glycoprotein in multidrug-resistant cell lines by monoclonal antibodies. *Nature*, **316**, 820.

KEMSHEAD, J.T., GOLDMAN, A., FRITZCHY, J., MALPAS, J.S. & PRITCHARD, J. (1983). Use of panels of monoclonal antibodies in the differential diagnosis of neuroblastoma and lymphoblastic disorders. *Lancet*, **i**: 2.

LAI, S.L., GOLDSTEIN, L.J., BOTTESMAN, M.M. & 7 others (1989). MDR1 gene expression in lung cancer. *J. Natl Cancer Inst.*, **81**, 1144.

MA, D.D.F., DAVEY, R.A., HARMAN, D.H. & 5 others (1987). Detection of a multidrug resistant phenotype in acute non-lymphoblastic leukaemia. *Lancet*, **i**, 135.

MERKEL, D.E., FUQUA, S.A.W., TANDON, A.K., HILL, S.M., BUZDAR, A.U. & MCGUIRE, W.L. (1989). Electrophoretic analysis of 248 clinical breast cancer specimens for P-glycoprotein overexpression or gene amplification. *J. Clin. Oncol.*, **7**, 1129.

MILLER, T.P., GROGAN, T.M., DALTON, W.S., SPIER, C.M., SCHEPER, R.J. & SALMON, S.E. (1991). P-glycoprotein expression in malignant lymphoma and reversal of clinical drug resistance with chemotherapy plus high-dose verapamil. *J. Clin. Oncol.*, **9**, 17.

NAKAGAWARA, A., KADOMATSU, K., SATO, S.-I. & 5 others (1990). Inverse correlation between expression of multidrug resistance gene and N-myc oncogene in human neuroblastomas. *Cancer Res.*, **50**, 3043.

OZOLS, R.F., CUNNION, R.E., KLECKER, W.R. Jr & 4 others (1987). Verapamil and adriamycin in the treatment of drug-resistant ovarian cancer patients. *J. Clin. Oncol.*, **5**, 641.

PHILIP, T., HELSON, L., BERNARD, J.L. & 4 others (1987). Definition of response and remission in children with advanced neuroblastoma: proposition for a scoring system. *Ped. Hemat. Oncol.*, **4**, 25.

PHILIP, T. & PINKERTON, R. (1989). Neuroblastoma. In Magrath, I. (ed.), *New Directions in Cancer Treatment*. Springer-Verlag, pp. 605-611.

ROTHENBERG, M. & LING, V. (1989). Multidrug resistance: molecular biology and clinical relevance. *J. Natl Cancer Inst.*, **81**, 907.

SCHLAIFER, D., LAURENT, G., CHITTAL, S. & 8 others (1990). Immunohistochemical detection of multidrug resistant associated P-glycoprotein in tumour and stromal cells of human cancers. *Br. J. Cancer*, **62**, 177.

SUGAWARA, I., KODO, H., OHKOCHI, E., HAMADA, H. & MORI, S. (1989). High-level expression of MRK 16 and MRK 20 murine monoclonal antibody-defined proteins (170,000–180,000 P-glycoprotein and 85,000 protein) in leukaemias and malignant lymphomas. *Br. J. Cancer*, **60**, 538.

THIEBAUT, F., TSURUO, T., HAMADA, H., GOTTESMAN, M.M., PASTAN, I. & WILLINGHAM, M.C. (1989). Immunohistochemical localization in normal tissues of different epitopes in the multidrug transport protein P170: evidence for localization in brain capillaries and crossreactivity of one antibody with a muscle protein. *J. Histochem. Cytochem.*, **37**, 159.

WEINSTEIN, R.S., KUSZAK, J.R., FLUSKENS, L.F. & COON, J.S. (1990). P-glycoproteins in pathology: the multidrug resistance gene family in humans. *Hum. Pathol.*, **21**, 34.